# Dog Ownership and Risk for Alveolar Echinococcosis, Germany

**DOI:** 10.3201/eid2808.212514

**Published:** 2022-08

**Authors:** Julian Schmidberger, Janne Uhlenbruck, Patrycja Schlingeloff, Pavlo Maksimov, Franz J. Conraths, Benjamin Mayer, Wolfgang Kratzer

**Affiliations:** University Hospital Ulm, Ulm, Germany (J. Schmidberger, J. Uhlenbruck, P. Schlingeloff, W. Kratzer);; Friedrich-Loeffler-Institute, Federal Research Institute for Animal Health, Greifswald-Insel Riems, Germany (P. Maksimov, F.J. Conraths);; Institute for Epidemiology and Medical Biometry, Ulm (B. Mayer)

**Keywords:** Alveolar echinococcosis, Echinococcus multilocularis, risk factor, dog ownership, case-control study, tapeworm, parasites, Germany

## Abstract

Human alveolar echinococcosis is caused by the parasite *Echinococcus multilocularis*, and dog ownership has been identified as a risk factor. We sought to specify the factors of dog ownership underlying this risk by conducting a case–control study among dog owners in Germany. The analysis revealed an increased odds ratio of ≈7-fold for dog owners whose dogs roam unattended in fields, 13-fold for dog owners who feed their dogs organic waste daily, 4-fold for dog owners who take their dog to a veterinarian only in case of illness, and 10-fold for dog owners who have never been informed by a veterinarian about the risk for infection. The results highlight the risk for infection associated with various factors of dog ownership and the value of veterinarians informing owners about prevention.

Human alveolar echinococcosis is a rare disease that can be caused by the parasite *Echinococcus multilocularis* (*1*,*2*). The pathogen *E. multilocularis* and human cases of the disease are predominantly distributed in the northern hemisphere (*3*,*4*). The most heavily affected countries in central Europe include Germany, France, Switzerland, and Austria (*1*,*5*), but large parts of Russia and China are also affected (*1*,*5*). Approximately 70%–80% of human cases in Germany are distributed in the main *E. multilocularis*–endemic areas of Baden-Württemberg and Bavaria. High-risk areas are found in the area of the Swabian Alb, the Alps, and the Alpine foothills (*6*). The prevalence of *E. multilocularis* infections in foxes in those areas is 40%–60% (*7*). 

The life and development cycle of *E. multilocularis* parasites involves definitive and intermediate hosts. Adult *E. multilocularis* parasites usually colonize the small intestine of carnivores, mostly red foxes (*Vulpes vulpes*), dogs, and cats (*2*,*8*,*9*). These hosts excrete infectious worm eggs into the environment in their feces, through which small mammals, such as field mice (*Microtus arvalis*), voles (Arvicolinae), bank voles (*Myodes glareolus*), and other species can become infected. In the intermediate hosts, larval *E. multilocularis* stages (metacestodes) usually grow in the liver, where they cause alveolar echinococcosis and travel with the blood or lymph to other organs, behaving similarly to malignant tumors. In this process, the parasite can irreversibly damage the organs of the intermediate host, which can lead to death (*1*,*8*,*9*). A diseased intermediate host represents easier prey for the final host, because of its disease manifestations and symptoms, closing the development cycle. Humans can be terminal intermediate hosts who, similar to other intermediate hosts, inadvertently ingest worm eggs (fecal–oral route) and produce metacestodes. In >98% of cases of human infection, the liver is the primary organ affected (*2*).

Risk factors for human alveolar echinococcosis have so far been incompletely investigated. The currently available case–control studies of risk factors are relatively old or cannot be applied to the situation in Germany (*10*–*13*). Studies in France, Austria, and Alaska (USA), suggest that dog ownership is one of the most significant risk factors for infection with *E. multilocularis* and development of alveolar echinococcosis (*11*–*13*). A case–control study conducted in Germany in 2004, involving 40 patients and 120 controls, found increased odds ratios (ORs) for owners of dogs that poach and run unattended outdoors; persons who live close to fields, live in a farmhouse, farm, chew grass, and gather wood; and cat owners (*10*). To date, factors that could not be confirmed as significant include eating unwashed strawberries, picking berries far from the ground, and collecting mushrooms. A meta-analysis considered those risk factors, including dog ownership (*14*). A systematic review and meta-analysis, in which 28 cross-sectional studies and 14 case–control studies were analyzed, also showed strong evidence for transmission by direct contact with dogs (*15*).

Data from the National Echinococcosis Registry Germany (https://www.fuchsbandwurm.eu), based on 673 patients with alveolar echinococcosis recorded during 1992–2018, show that 60%–75% of recorded patients own, have owned, or have had regular contact with >1 dogs. In Germany, according to a joint survey by the Central Association of Pet Owners (Zentralverband Zoologischer Fachbetrieb, https://www.zzf.de) and the Pet Supplies Industry Association (Industrieverband Heimtierbedarf, https://www.ivh-online.de), an estimated 9.4 million dogs lived in 19% of households in 2018. This estimate represents an increase of 2 million dogs since 2011 (*16*,*17*). Our aim with this case–control study was to further specify and examine in more detail the factors of dog ownership that are potential risk factors for human alveolar echinococcosis. 

## Methods

### Study Design

**Figure 1:**
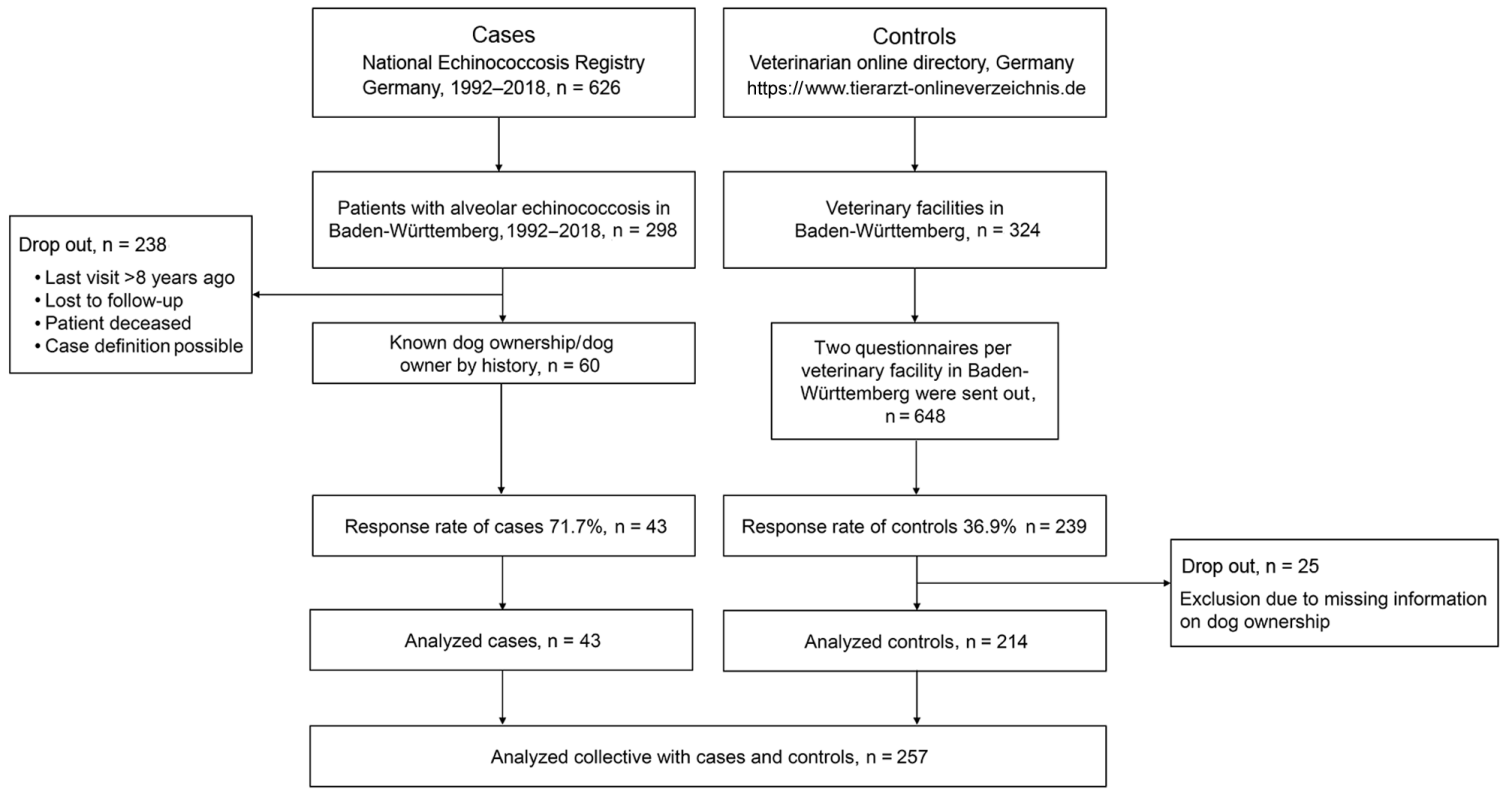
Inclusion and exclusion process for 43 case-patients and 214 controls in case–control study of dog ownership and human risk for alveolar echinococcosis, Baden-Württemberg, Germany, January 2019–February 2020.

**Figure 2:**
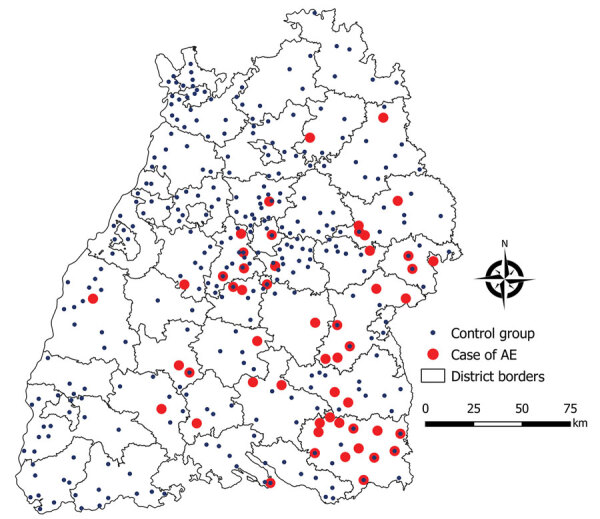
Distribution of 60 alveolar echniococcosis case-patients and 324 controls, Baden-Württemberg, Germany, January 2019–February 2020.

For this case–control study, we recruited patients with alveolar echinococcosis from the National Echinococcosis Registry Germany and recruited healthy volunteers from veterinary and veterinary medical facilities listed in the Veterinary Online Directory Germany (Figures 1, 2). We conducted a written survey of case-patients and controls during January 2019–February 2020 by using a questionnaire with 45 questions with dichotomous expressions and a 3-5–point Likert scale prepared for this purpose. The questionnaire included general questions about the dog (e.g., breed, coat length, sex) and its dietary behavior, deworming, grooming, and cleaning, as well as human dog-ownership habits.

### Alveolar Echinococcosis Case-Patients

The National Echinococcosis Registry Germany is a national disease registry that is part of a Deutsche Forschungsgemeinschaft–funded project in cooperation with the Robert Koch Institute (*6*). The registry records on a voluntary basis all cases of the disease diagnosed in Germany since 1992 (n = 626 as of December 31, 2018). Compared with the cases reportable to the Robert Koch Institute within the framework of the reporting obligation according to the Infection Protection Act (https://www.gesetze-im-internet.de), cases in the National Echinococcosis Registry Germany include extensive information on epidemiology, risk factors, diagnostics, treatments, and patient care. In accordance with the study design, we selected cases from Baden-Württemberg, an area in southern Germany where alveolar echinococcosisis is highly endemic. 

**Figure 3:**
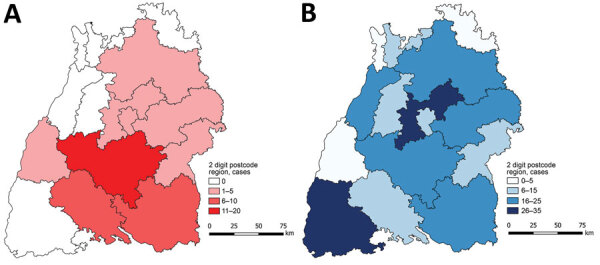
Choropleth map showing the distribution and frequency of 43 patients with alveolar echinococcosis (A) and 214 controls (B) who participated in case–control study of dog ownership and human risk for alveolar echinococcosis, by 2-digit postal code region, Baden-Württemberg, Germany, January 2019–February 2020.

During 1992–2018, a total of 298 case-patients residing in Baden-Württemberg were registered in the national disease registry. Of these, we recruited 60 case-patients who, according to their medical history, owned dogs. Case-patients were excluded if their last visit to the hospital was >8 years earlier, if they had died, or if their case definition was only possible according to World Health Organization–Informal Working Group on Echinococcosis criteria (*2*) or no information on dog ownership was available (n = 238 excluded patients). From the 60 contacted case-patients, we received 43 completed questionnaires from 43 dog owners with alveolar echinococcosis, resulting in a response rate of 71.7% (Figure 3, panel A).

### Control Group

The Veterinary Online Directory Germany (https://www.tierarzt-onlineverzeichnis.de) includes all veterinary facilities in Germany registered on a voluntary basis. We identified 324 veterinary facilities for Baden-Württemberg. We sent 2 questionnaires to each veterinary facility in Baden-Württemberg (n = 648) and distributed them to dog owners without known alveolar echinococcosis. We received completed questionnaires back from 239 dog owners, resulting in a response rate of 36.9%. Of these questionnaires, we used 214 in the final analysis (Figure 3, panel B) and excluded 25 because of missing information about dog ownership.

### Statistical Analyses

For statistical analyses, we used SAS version 9.4 (https://www.sas.com). We initially analyzed the data descriptively. We determined mean values ±SDs and median, minimum, and maximum values and presented them as absolute or relative frequencies. For variables that may influence the occurrence of alveolar echinococcosis, we used a multivariable logistic regression model to determine adjusted odds ratios (aORs), 95% CIs, and p values. Confounding variables determined a priori were included in the adjusted logistic regression model. We used Pearson χ^2^ and Fisher exact tests to identify possible relationships and differences in frequency distributions between dichotomous variables. The significance level was set at α = 0.05. Dot-density maps and choropleth maps of cases and controls and their distributions were created by using the QGIS geographic information system version 3.16.0 (https://www.qgis.org).

### Ethics Statement

The study was approved by the local ethics committees of the University of Ulm (approval no. 125/20) and conducted according to the Declaration of Helsinki. Written informed consent was obtained from all case-patients and controls.

## Results

The study included 43 persons with cases that fit the World Health Organization case definition of confirmed or probable (case-patients) and 214 controls from Baden-Württemberg. The mean age of the case-patients was 50.09 ± 17.62 years and of controls was 44.23 ± 15.23 years (p = 0.0347). The proportion of women was significantly lower among case-patients (26/43 [60.47%]) than controls (177/214 [82.71%]) (χ^2^ = 10.6757; p = 0.0011). A total of 41/43 (95.35%) case-patients and 182/214 (85.05%) controls reported that they had lived in Baden-Württemberg for >20 years (p = 0.0839 by Fisher exact test) (Table 1). The main country of origin is Germany for dogs owned by case-patients 21/24 (87.50%) and 89/128 (69.53%) for dogs owned by controls (p = 0.08417 by Fisher exact test). No information was available for the origin of the dogs for 19/43 (44.19%) of the case-patients and 86/214 (40.19%) of the controls.

**Table 1:** Characteristics of 257 dog owners with alveolar echinococcosis and healthy controls in case–control study of dog ownership and human risk for alveolar echinococcosis, Baden-Württemberg, Germany, January 2019–February 2020*

Characteristic	Case-patients, n = 43	Controls, n = 214	p value
Sex, no. (%)			**0.0011**
F	26 (60.47%)	177 (82.71%)	
M	17 (39.53%)	37 (17.29%)	
Age, y, no. (%)			**<0.0001**
18–30	8 (18.60%)	56 (26.17%)	
31–50	13 (30.23%)	71 (33.18%)	
51–70	15 (34.88%)	80 (37.38%)	
>70	7 (16.28%)	7 (3.27%)	
Age, y			**0.0347**
Mean ± SD	50.09 ± 17.62	44.23 ± 15.23	
Median (range)	51.00 (18.00–79.00)	44.00 (19.00–88.00)	
Time lived in Baden-Württemberg, y			0.0839
5–20	2 (4.65%)	32 (14.95%)	
>20	41 (95.35%)	182 (85.05%)	
Dogs owned, no. (%)			0.0546
1	32 (74.42)	122 (57.01)	
2	6 (13.95)	56 (26.17)	
3	2 (4.65)	22 (10.28)	
>3	3 (6.98)	14 (6.54)	
Regular contact with dogs, y, no. (%)			0.2629
0–5	4 (9.30)	16 (7.48)	
6–10	2 (4.65)	34 (15.89)	
11–20	10 (23.26)	48 (22.43)	
>20	27 (62.79)	116 (54.21)	
*Boldface indicates significance (p<0.05).			

### Dog Ownership 

The duration of dog ownership and regular contact with dogs was >20 years for 27/43 (62.79%) of the case-patients and 116/214 (54.21%) of the controls. The duration of dog ownership and regular contact with dogs was 11–20 years for 10/43 (23.26%) of the case-patients and for 48/214 (22.43%) of the controls. Thus, duration of dog ownership and regular contact with dogs did not differ significantly between the groups (χ^2^ = 1.2533; p = 0.2629).

The number of dogs owned also did not differ significantly between the groups (χ^2^ = 3.6938: p = 0.0546) but was remarkably discrepant between the groups (Table 1). In the case group, the proportion of dog owners with 1 dog was 32/43 (74.42%), and in the control group it was 122/214 (57.01%). The survey further revealed that 6/43 (13.95%) of the case-patients and 56/214 (26.17%) of the controls owned 2 dogs. The percentage of dog owners with >3 dogs was 3/43 (6.98%) for case-patients and 14/214 (6.54%) for controls (Table 1).

### Risk Behavior and Habits of Dog Owners

The aOR for alveolar echinococcosis increased by ≈7-fold for owners whose dogs roamed unattended in fields compared with owners whose dogs roamed unattended in yards (aOR 7.081, 95% CI 1.523–32.931; p = 0.0126) (Table 2). For dog owners whose dogs rarely rolled in other animals’ feces, odds of acquiring alveolar echinococcosis were lower (aOR 0.205, 95% CI 0.078–0.538; p = 0.0013). Furthermore, the analysis revealed a nearly 13-fold increase in OR for alveolar echinococcosis among dog owners who fed their dogs organic waste daily (aOR 12.840, 95% CI 1.127–146.278; p = 0.0398) (Table 2). ORs were potentially increased but not statistically significant for dog owners from rural communities (aOR 4.175, 95% CI 0.711–24.534, p = 0.9559) and those whose dogs ate carrion or rodents (aOR 2.125, 95% CI 0.542–8.340, p = 0.2798). Odds increased 7-fold for those who owned a herding dog (aOR 6.831 95%, CI 1.028–45.371), and having a dog with an undercoat seemed to be significantly protective (aOR 0.319, 95% CI 0.102–0.997) (Table 2).

**Table 2:** Multivariable logistic regression analysis with estimations of odds of acquiring alveolar echinococcosis, Baden-Württemberg, Germany, January 2019–February 2020*

Variable	Alveolar echinococcosis	
	aOR (95% CI)	p value†
Community type, no. residents		
Large city, >100,000	Referent	
Middle city, 20,000–100,000	0.127 (0.009–1.789)	0.1263
Small city, 5,000–20,000	1.054 (0.161–6.917)	0.9559
Rural community, <5,000	4.175 (0.711–24.534)	0.9559
Unattended		
Garden	Referent	
Field	7.081 (1.523–32.931)	**0.0126**
Forest	1.221 (0.362–4.120)	0.7473
Dog eats carrion or prey		
Frequently	2.125 (0.542–8.340)	0.2798
Rarely	0.514 (0.233–1.135)	0.0998
Never	Referent	
Dog rolls in feces from other animals		
Frequently	2.570 (0.962–6.865)	0.0598
Rarely	0.205 (0.078–0.538)	**0.0013**
Never	Referent	
Dog hunts mice or prey		
Frequently	0.664 (0.242–1.821)	0.4260
Rarely	0.766 (0.334–1.756)	0.5292
Never	Referent	
Dog eats organic waste from other animals		
Daily	12.840 (1.127–146.278)	**0.0398**
Weekly	<0.001 (<0.001–>999.999)	0.9843
Monthly	<0.001 (<0.001–>999.999)	0.9908
Never/ sporadic	Referent	
Frequency of fur cleaning		
Daily	Referent	
Weekly	0.472 (0.163–1.368)	0.1665
Monthly	0.400 (0.075–2.140)	0.2840
When soiled	0.543 (0.216–1.366)	0.1945
Never	7.567 (0.655–87.406)	0.1050
Veterinary visits		
Only in case of illness	3.657 (1.480–9.039)	**0.0050**
1 vist/y	2.003 (0.767–5.233)	0.1560
>1 visit/y	Referent	
Deworming frequency		
1 time/mo	Referent	
3–4 times/y	0.183 (0.031–1.061)	0.0582
1 time/y	0.799 (0.125–5.091)	0.8124
If infection is suspected	0.190 (0.022–1.599)	0.1264
Never	0.734 (0.064–8.437)	0.8040
Feces tested for worm eggs		
Regularly	Referent	
If infection is suspected	0.225 (0.045–1.118)	0.0681
Never	2.262 (0.598–8.562)	0.2292
Owner received education from veterinarian		
Yes	Referent	
No	10.006 (4.282–23.383)	**<0.0001**
Purpose of dog ownership		
Hunting	0.332 (0.083–1.329)	0.1192
Herding	6.831 (1.028–45.371)	**0.0467**
Sporting	0.668 (0.077–5.808)	0.7148
Guard/watch dog	2.776 (0.819–9.412)	0.1011
Breeding	<0.001 (<0.001–>999.999)	0.1011
Pet	Referent	
Other	2.132 (0.149–30.566)	0.5773
Coat length of the dog		
Short, 1–2 cm	Referent	
Medium, 2–7 cm	0.902 (0.371–2.192)	0.8205
Long, >7 cm	0.709 (0.205–2.446)	0.5860
Undercoat	0.319 (0.102–0.997)	**0.0493**

*Model adjusted for age and sex. Boldface indicates significance (p<0.05).

### Dog Cleaning and Prevention Behavior

Multivariable logistic regression adjusted for age and sex revealed an almost 4-fold increased odds ratio for dog owners who took their dog to a veterinary facility only for illness compared with dog owners who sought veterinary care >1 time/year (aOR 3.657, 95% CI 1.480–9.039; p = 0.0050). For dog owners who never received information from a veterinarian about their own risk for *E. multilocularis* infection and possible prevention, the analysis further revealed a 10-fold increase in odds for alveolar echinococcosis (aOR 10.006, 95% CI 4.282–23.383; p<0.0001) (Table 2).

In contrast, for dog owners who never had their dog’s feces tested for worm eggs, the odds for alveolar echinococcosis were not significantly increased (aOR 2.262, 95% CI 0.598–8.562; p = 0.2292) (Table 2). Odds were increased by 7-fold for dog owners who never cleaned their dog’s coat compared with dog owners who cleaned their dog’s coat daily (aOR 7.567, 95% CI 0.655–87.406; p = 0.1050), but the difference was not statistically significant. Furthermore, odds were not significantly increased between case-patient and control groups with regard to dog deworming (p>0.05).

## Discussion

Our case–control study of the potential contributions of factors of dog ownership to the risk for human alveolar echinococcosis in Baden-Württemberg was based on the findings of previous case–control studies and systematic reviews that described dog ownership as an evident risk factor for human alveolar echinococcosis (*10*–*15*). We found a significantly increased risk for alveolar echinococcosis among dog owners whose dogs roamed unattended in fields. Other factors that may increase risk are ownership of dogs in rural communities, dogs that roll in the feces of other animals, and dogs that eat carrion or prey.

Kern et al. demonstrated increased risk for owners of dogs that poach, owners of dogs that run unattended outdoors, and persons who live close to a field (*10*). Those results and ours are consistent with the fact that in the rural areas of the study region, there is a potential reservoir of the parasite in wildlife populations, particularly foxes (definitive hosts) and their natural prey (i.e., small mammals) (*18*). The overall risk may be elevated by increased environmental contamination with *E. multilocularis* eggs, including increased prevalence of dog feces in the environment.

Studies suggest that the prevalence of *E. multilocularis* parasites in dogs as the final host can vary greatly, depending on the study region, and may play a subtantial role in transmitting the pathogen (*19*–*25*). In rural areas, such as southern Germany, dogs that roam unattended in fields and prey on rodents have an increased chance of injesting infected prey and thus enabling completion of the parasite’s life and development cycle. If dogs then excrete worm eggs, it is plausible that their owners’ risk of contracting alveolar echinococcosis is increased. However, the general risk for persons living in rural, echinococcosis-endemic areas is also likely to be increased because of the higher level of environmental contamination with *E. multilocularis* eggs.

We found also a significantly increased risk for alveolar echinococcosis for dog owners who took their dog to a veterinarian only if it was ill, who had never been informed by a veterinarian about their own risk for infection with the fox tapeworm *E. multilocularis*, and who dewormed their dog(s) infrequently. These results seem plausible because studies have shown that dogs in rural areas where risk for infection is higher receive less veterinary care (*8*,*22*) and that dogs in rural areas, specifically unattended dogs, are more likely to be infected (*21*). Studies of dog feces from different countries show *E. multilocularis* infestation rates of 1.5%–20%, depending on the study setting (*19**–**25*). PCR analysis of 21,588 dog feces samples collected during 2004–2005 in Germany indicated an overall *E. multilocularis* prevalence of 0.24% (43/17,894); prevalence was higher in southern Germany (0.35%, 31/8,941) than in northern Germany (0.13%, 12/8,953) (*26*). The authors estimated that the chance of a dog becoming infected with the parasite within 10 years was 8.7%. Knapp et al. found that the high occurrence of dog feces in the cities studied, despite a lower prevalence of *E. multilocularis* infection, posed a clear risk for humans (*27*). Accordingly, dogs kept for private reasons were less likely to be carriers of the parasite (<1.5%) than were herding or hunting dogs that had free range and hunted rodents (3%–8%). Towes et al. also found higher proportions of infected animals among hunting and herding dogs that were allowed to roam freely (*21*), as did we in our study.

Strube et al. argued that, depending on the active ingredient and anthelmintic, 62.4% (312/500) of dogs should be dewormed 12 times per year, according to European Scientific Counsel Companion Animal Parasites (*28*). Those guidelines also said that another 30.8% (154/500) of the dogs studied should be dewormed according to category C, 4.8% (24/500) 4 times/year and 2.0% (10/500) 1 or 2 times/year. The study showed poor deworming practices, with an average of only 2.07 dewormings/year. Dog fur contaminated with *E. multilocularis* eggs may be another source of transmission to humans (*29*). That possibility contrasts with our finding that a dog having an undercoat is protective. Given the denseness of this coat structure, the possibility of transmission can be assumed. The study by Nagy et al. shows that contamination via the coat is possible, which in turn means that inadequate coat care may be associated with increased risk for disease, as our study suggests (*29*). Thus, lack of education and knowledge about the potential risk for infection with *E. multilocularis* parasites and poor canine hygiene/grooming may be associated with higher odds of possible human infection.

We do not have a plausible explanation for the increased risk for dog owners who feed dogs organic waste on a daily basis. Possibly this feeding behavior is confounded by the behavior of dogs with a certain purpose (e.g., hunting, herding, and guarding [watch dogs]).

Limitations of our study are the heterogeneous distribution of case-patients and controls, as well as possible recall bias. Because patients with alveolar echinococcosis and the risk factor of dog ownership in Baden-Württemberg are in a highly selected, heterogeneously distributed group, this potential confounding factor may be overcome only by conducting an international study, perhaps in a multicentered format. Because of the small number of cases and often heterogeneous group sizes, values may scatter substantially, and CIs can vary widely. 

Our study shows that certain factors of dog ownership are associated with increased odds of human alveolar echinococcosis and provides an overview of other potential risk factors. Considering the rapidly increasing number of dog owners in Germany, the results emphasize the role of veterinary facilities and others in informing dog owners about preventing or reducing their risk for infection with *E. multilocularis* parasites.
